# Equity in utilization of antiretroviral therapy for HIV-infected people in South Africa: a systematic review

**DOI:** 10.1186/s12939-014-0060-z

**Published:** 2014-08-01

**Authors:** Noor Tromp, Charlotte Michels, Evelinn Mikkelsen, Jan Hontelez, Rob Baltussen

**Affiliations:** 1 Department of Primary and Community Care, Radboudumc, Nijmegen, The Netherlands; 2 Department of Public Health, Erasmus MC, University Medical Center Rotterdam, Rotterdam, The Netherlands

**Keywords:** Antiretroviral therapy, Equity, South Africa, Systematic review

## Abstract

**Introduction:**

About half a million people in South Africa are deprived of antiretroviral therapy (ART), and there is little systematic knowledge on who they are – e.g. by severity of disease, sex, or socio-economic status (SES). We performed a systematic review to determine the current quantitative evidence-base on equity in utilization of ART among HIV-infected people in South Africa.

**Method:**

We conducted a literature search based on the Cochrane guidelines. A study was included if it compared for different groups of HIV infected people (by sex, age, severity of disease, area of living, SES, marital status, ethnicity, religion and/or sexual orientation (i.e. equity criteria)) the number initiating/adhering to ART with the number who did not. We considered ART utilization inequitable for a certain criterion (e.g. sex) if between groups (e.g. men versus women) significant differences were reported in ART initiation/adherence.

**Results:**

Twelve studies met the inclusion criteria. For sex, 2 out of 10 studies that investigated this criterion found that men are less likely than women to utilize ART, while the other 8 found no differences. For age, 4 out of 8 studies found inequities and reported less utilization for younger people. For area of living, 3 out of 4 studies showed that those living in rural areas or certain provinces have less access and 2 out of 6 studies looking at SES found that people with lower SES have less access. One study which looked at the marital status found that those who are married are less likely to utilize ART. For severity of disease, 5 out of 6 studies used more than one outcome measure for disease stage and reported within their study contradicting results. One of the studies reported inconclusive findings for ethnicity and no study had looked at religion and sexual orientation.

**Conclusion:**

It seems that men, young people, those living in certain provinces or rural areas, people who are unemployed or with a low educational level, and those being unmarried have less access to ART. As studies stem from different contexts and use different methods conclusions should be taken with caution.

## Introduction

South Africa is home to the largest HIV-infected population worldwide, with 6.1 million people living with HIV/AIDS in 2012 [1]. The country also has the largest antiretroviral therapy (ART) program worldwide: with domestic investments amounting to US$1.9 billion in 2011 [2], it provided treatment to about 80% (2.0 million people) of all eligible people in 2012 [1]. Current South African guidelines state that all those with CD4 cell counts of ≤350 cells/*μ*L are eligible for ART [3].

Nevertheless, a significant treatment gap of about half a million people remains between those who receive treatment and those in need according to the eligibility criteria [1]. There is little knowledge on which people are deprived from treatment – e.g. by severity of disease, sex, age, socio-economic status (SES) and area of living [4], limiting the development of policy measures to specifically target and improve treatment coverage among these groups. This is illustrated in South Africa’s ‘National Strategic Plan on HIV, STIs and TB 2012-2016’ which flags the importance of inequalities in treatment utilization but is not specific on which marginalized groups should be targeted [5].

It is clear that ART not only improves a patient’s health and survival [6],[7], but also substantially reduces their infectiousness [8],[9]. As a result, ART can play an important role in controlling the epidemic in South Africa [10]-[12]. The World Health Organization (WHO) recently released new consolidated guidelines, taking both the prevention and treatment benefits of ART into account [13],[14]. The new guidelines state that ART should be provided for HIV infected people with a CD4 cell count of ≤500 cells/μL, who are in a serodiscordant relationship, and/or pregnant [14]. In addition, the WHO also states that guidelines should be expanded when universal access for those with CD4 cell counts of ≤350 cells/μL has already been achieved [14]. As treatment programs continue to expand, identifying and targeting hard-to-reach populations will be increasingly important.

We determined the current quantitative evidence-base on equity in utilization of antiretroviral therapy (ART) among HIV-infected people in South Africa. This information may provide insight into methods used for equity research and may help policy makers to identify and target hard-to-reach populations.

## Methods

We performed a systematic review on the basis of the Cochrane Handbook for Systematic Reviews of Interventions, Version 5.1.0.4 [15]. Our search was performed on 18 February 2013 using Pubmed, Embase, Central and Psychinfo database. Our search syntax consisted of search terms in four categories (ART, HIV, South Africa and Equity), that were combined using AND. The search strategy is presented in summary in Table 1 and in detail in Additional file 1.

**Table 1:** Search strategy employed in systematic review of studies on equity in ART utilization in South Africa

**Category**	**Search terms (in Pubmed database)**
*ART*	Antiretroviral therapy, highly active [MeSH Terms] OR ART [title/abstract] OR HAART [title/abstract] OR AR V [title/abstract] OR ARVs [title/abstract] OR Anti-Retroviral Agents [Mesh] OR antiretroviral [title/abstract] OR anti retroviral [title/abstract] OR anti-retroviral [title/abstract] OR antiviral [title/abstract] OR therapy [title/abstract]
	AND
*HIV*	Acquired immunodeficiency syndrome [MeSH Terms] OR acquired immunodeficiency syndrome [title/abstract] OR aids [title/abstract] OR hiv [MeSH Terms] OR hiv [title/abstract] OR human immunodeficiency virus [title/abstract] OR HIV infections [MeSH Terms]
	AND
*South Africa*	(South Africa [MeSH Terms] OR (South [title/abstract] AND Africa* [title/abstract]))
	AND
*Equity*	(Equity [title/abstract] OR equities [title/abstract] OR inequity [title/abstract] OR inequities [title/abstract] OR equality [title/abstract] OR equalities [title/abstract] OR equal [title/abstract] OR equitable [title/abstract] OR inequality [title/abstract] OR inequalities [title/abstract] OR unequal [title/abstract] OR disparity [title/abstract] OR disparities [title/abstract] OR vulnerability [title/abstract] OR fairness [title/abstract] OR unfair [title/abstract] OR social justice [MeSH Terms] OR social justice [title/abstract] OR justice [title/abstract] OR barrier [title/abstract] OR coverage [title/abstract] OR barriers [title/abstract] OR healthcare disparities [MeSH Terms] OR health services accessibility [MeSH Terms] OR health services accessibility [title/abstract] OR access to health care [title/abstract])

### Conceptual model

Following the WHO’s guidance on monitoring equity in AIDS treatment programs [16], we distinguished five domains of coverage: 1) availability of resources; 2) physical and financial accessibility; 3) acceptability; 4) use of service; and 5) effective coverage (defined as the proportion of the population in need of an intervention who fully comply with the recommended treatment program). This review focuses on the latter two domains as the other domains feed into these. We included studies on both ART initiation and adherence and this was together labeled as ‘ART utilization’. We acknowledge that an individual’s health care utilization can be explained by a function of predisposing factors (e.g. education, culture, health beliefs, age and sex), enabling factors (income, health insurance, waiting time, genetic factors) and need factors (perceived need to seek and adhere to care and professional’s judgment about people’s health status) [17]. We used the terms ‘equity’ and ‘inequity’ to reflect differences in utilization of ART by criteria such as severity of disease, age, or SES [18].

### Inclusion and exclusion criteria

A study was included if it: 1) compared for different groups of HIV infected people (by sex, age, severity of disease, area of living, socio-economic status, marital status, ethnicity, religion and/or sexual orientation (i.e. equity criteria (World Health Organization, Guidance on Priority Setting in Health Care (GPS health) in preparation) [19]) the number initiating/adhering to ART with the number who are not); 2) was performed in South Africa; and 3) reported in English. Although some equity criteria are the social determinants of health, severity of disease is not and therefore we preferred to use the term ‘equity criteria’ which was put forward by the WHO [World Health Organization, Guidance on Priority Setting in Health Care (GPS health) in preparation] and Tromp *et al.*[19]. A study was excluded if it: 1) focused on prevention of mother to child transmission (PMTCT), death during follow up, barriers of accessing care or tuberculosis (TB) services for HIV infected patients; 2) was a qualitative study, comment, editorial, economic evaluation or conference abstract; 3) was a duplicate reference from different databases; and 4) reported only differences in groups by a simple comparison with the gross number of people initiating or adhering to ART. We only included studies that take into account the underlying need of a group for ART. For example, the mere fact that more women than men have access to ART does not necessarily indicate an inequity as more women than men may be infected in the country. There was no restriction for publication date for inclusion of studies. Following the Cochrane guidelines grey literature was excluded due to expected low methodological quality of studies [15].

### Study selection, data extraction and quality evaluation

Two independent reviewers (CM and EM) assessed if the studies from the database search satisfied the inclusion criteria. First, all studies were screened on the basis of title and abstract, and subsequently on the basis of full-text. Reference lists of the retrieved articles were screened for additional studies (snowballing). The reviewers used a data collection form (Additional file 2) to extract relevant information (study characteristics, results per equity criteria, and study limitations) from the articles. Both reviewers evaluated the quality of studies using a quality-grading protocol (Additional file 2) adapted from existing protocols [15],[20],[21]. The protocol covers 20 indicators and for each item 0–2 points are given and added up to get an overall quality score (ranging from 0 to 40 points). Studies were categorized as low-quality (<20 points), medium-quality (20–29) or high-quality (≥30). During the study selection, data extraction and quality assessment, disagreements were resolved through discussion with a third researcher (NT) until consensus was reached.

### Data synthesis and analysis

A matrix was developed containing the study results per investigated equity criterion. We established the following categories to summarize the results for each equity criteria investigated in a study: 1) *associated,* differences reported in ART utilization between groups (e.g. men versus women for sex) were significant (p < 0.05, or when 1.0 does not fall in 95% confidence interval (95% CI)); 2) *not associated* differences reported in ART utilization between groups were not significant (p value >0.05 or 1.0 falls in 95% CI; *contradicting results,* within one study contradicting results were reported for differences in ART utilization between groups due to the use of multiple outcome measures for an equity criterion (e.g. CD4 cell count levels and WHO disease stages for the equity criterion severity of disease); and *inconclusive results,* differences in ART utilization between groups was investigated but the authors drew no conclusions due to small sample sizes.

We adhered to the PRISMA guidelines for reporting of this systematic review [22].

## Results

### Study inclusion

**Figure 1:**
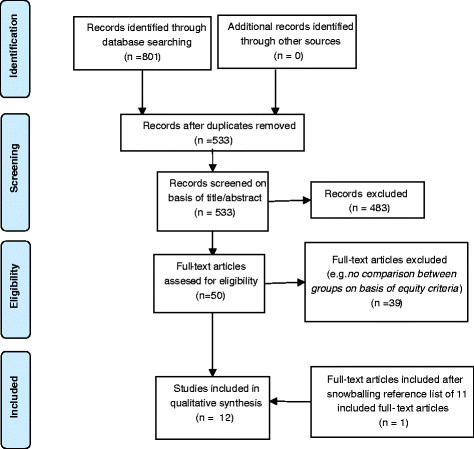
‘Flow diagram showing study selection for systematic review of studies on access to antiretroviral therapy in South Africa’.

From the initial search (801 articles), 268 studies were duplicates, 483 studies were excluded on the basis of title/abstract and 39 on the basis of full-text screening. Screening of the references of the remaining 11 studies resulted in one extra article and added to a total of 12 studies that are included in this review (Figure 1, Table 2).

**Table 2:** Overview of reported findings per study on association between equity criteria and ART initiation or adherence

**Reference**		***Equity criteria***						
**Name, year**	**Quality score**	**Sex**	**Age**	**Severity of disease**	**Area of living**	**Socio-economic status (including education and employment)**	**Marital status**	**Ethnicity**
***ART initiation*** *(lower < higher likelihood to initiate)*								
**Cleary 2011**[25]	***	**Not associated,** men = women				*SES:***not associated,** SES distribution HIV + in need = ART clinic patients		**Inconclusive results** population size too small to draw conclusions
**Cooke 2010**[32]	***	**Not associated**						
		men = women	**Associated,** younger (15–19 yrs), < older (>19 yrs)		**Not associated,** peri-urban = urban = rural	*SES:***not associated,** index profile 1 = 2 = 3 = 4 = 5 (SES), *education:***not associated**, years of education as continuous variable		
**Govindasamy 2011**[26]	***	**Not associated,** men = women	**Not associated,** ≤ 30 yrs = ≥ 30 yrs	**Contradicting results,***CD4 cell count:***associated,** CD4 > 350 < ≤350, *WHO stages:***not associated**		*Education:***not associated,** primary school completed = not completed, *employment:***not associated,** employed < unemployed		
**Tsai 2009**[33]	**	**Not associated,** men = women	**Associated,** younger (18–30 yrs) < older (30–35 yrs)			*Education:***associated,** lower education (secondary) < higher education (matric/ tertiary), *employment:***associated,** non salaried employment < salaried employment, unemployed < employed	**Associated** never married < married or cohabiting	
**Adam 2009**[34]	**				**Associated**^**4**^, unequal ART coverage between 9 provinces			
**Muula 2007**[24]	*	**Associated**^**4**^, male < female						
**Nattrass 2006**[23]					**Associated**^**4**^, unequal ART coverage between 9 provinces			
***ART adherence*** *(poorer < better adherence)*								
**Boyles 2011**[30]	***	**Not associated,** men = women	**Associated,** younger (<25 yrs) < older, (25–50 yrs)	**Associated,** CD4 ≥ 200 < <200				
**Orrell 2003**[31]	***	**Not associated,** men = women	**Associated,** younger < older, adherence group is older (34 yrs) than non-adhererce group (31 yrs)	**Contradicting results,***CD4 cell count:***associated,** patients that not adhere had lower CD4 level, *Viral load:***associated,** patients that not adhere had higher VL, *WHO stage*: **not associated**		*SES:***not associated,** % low SES in patient group that continued, ART = that not continued		
**Kranzer 2010**[27]	***	**Associated,** men < women	**Not associated,** ≤ 30 yrs = > 30 yrs	**Contradicting results,***CD4 cell count:***associated,** > 200 < ≤100, *WHO stage:***not associated**				
**Fatti 2010**[29]	***	**Not associated,** men = women	**Not associated,** younger children (≤2 yrs) = older children (>2 yrs)	**Contradicting results,***CD4 cell count (severe immunodeficiency*^*1*^*):***associated,** patients with severe immunodeficiency were less adherent, *WHO stage (severe clinical status*^*2*^*):***not associated**	**Associated,** rural/urban < urban/urban < rural**/**rural^3^			
**Cornell 2009**[28]	**	**Not associated,** men = women	**Not associated,** age as continuous variable	**Contradicting results,***CD4 cell count:***associated**, CD4 < 50 < 50–150, but CD4 < 50 = >150, *WHO stage:***not associated**, *Viral load:***not associated**		*Employment:***associated,** no income < income		

ART = antiretroviral therapy, LTFU = lost to follow up, SES = socio-economic status, VL = viral load, WHO = world health organization.

^1^defined according to WHO criteria, i.e. < 12 months old: CD4 percentage <25% or CD4 count <1500 cells/mm^3^; 12–35 months: <20% or CD4 count <750; 36–59 months: CD4 percentage <15% or CD4 count <350; 5 years and older: CD4 count <200.

^2^defined as a WAZ score of < −3 (severe underweight) or a WHO stage ≥3.

^3^rural/urban, urban/urban, rural/rural = first term indicates place of residence and second the area of accessing ART clinic.

^4^no significance was reported and authors concluded that ART utilization was different among groups that were compared.

* = low quality, ** = medium quality, *** = high quality.

### Characteristics of included studies

Seven studies assessed inequities in ART initiation (Table 3) and five studies in ART adherence (Table 4). All studies were based on primary data analysis from observational surveys, except for one study using secondary data [23] and one review [24]. Studies defined ART initiation differently, like ‘at least 14 days on ART’ [25] or ‘visited the ART clinic at least once after testing HIV positive’ [26]. Definitions of non-adherence were also varied, and were measured in terms of patients’ absence at the clinic for more than one [27] three [28],[29] or six [30] months, or in terms of the number of pills not taken and brought back to the clinic (clinic-based pill counts) [31]. The outcome measure used for equity criteria varied widely among studies. For severity of disease, some compared the differences in utilization of ART by WHO disease stages [26]-[29],[31], while others used CD4 cell count levels [26]-[31] or viral load [28],[31]. For age, many different age categories were used. Fatti *et al.*[29] only included children in the study population, and the oldest age group in that sample is younger than the youngest age group in for example Govindasamy *et al.*[26] (who compared people below and above 30 years of age). Six studies, all using different databases, investigated urban and rural areas of the Western cape province and two studies reported at the national level [23],[24]. More than half of the articles (seven) [25]-[27],[29]-[32] were of high-quality, three had medium-quality [28],[33],[34] and two were of low-quality [23],[24]. Table 5 gives an overview of the quality scoring per study.

**Table 3:** Overview of finding per study reporting on equity in ART initiation

**Study, quality score, study type**	**Study objective**	**Study area, type of clinic/program**	**Year of data collection**	**Study design (comparison between population a and b), population sizes, sampling method/inclusion criteria**	**Statistical analysis**	**Outcome on association as reported per equity criteria**
Cleary 2011 *** Observational [25]	To evaluate whether the distribution of ART services in the public system reflects the distribution of people in need among adults in the urban population	**Urban area:** poor communities in Mitchells Plain (Cape Town, Western Cape province) and Soweto township (Johannesburg, Gauteng province), **public clinics**	National survey: 2008. Urban clinic data: unknown	**a. Population in need for ART (n = 742):** national survey (2008, HIV + residents), sampling unknown,	Comparison distribution of equity criteria (i.e. patients characteristics)	**Sex (not associated):** percentage of HIV + women in national survey is same as in ART users in urban clinic; 67.4% [95% CI: 61.5-72.9] versus 65.7% [95% CI: 60.6-70.7], p >0.05. **Socioeconomic status (not associated):** no significant differences in SES distribution between HIV + in need for ART and ART patients in urban clinics; independence partition Pearson’s chi-square test: 8 [p = 0.43] **Race/ethnicity (inconclusive results):** percentage of non-African is 2,5% in population HIV + in need versus 4.3% of ART users in urban clinics, authors state that sample size of non-African is too small to draw conclusions on equity
				**b. ART patients in urban public clinics (n = 635):** data from ART users (>18 yrs, >14 days on treatment) in three clinics in Mitchells Plain (selected proportional to the number of ART patients in facility) and three in Soweto (stratified random sampling)		
Cooke 2010 *** Observational [32]	To investigate factors associated with uptake of ART through a primary health care system in rural South Africa	**Rural, peri-urban and urban areas:** Hlabisa sub-district, Umkhanyakude district, Northern KwaZulu-Natal province, **public clinics supported by NGOs**	Aug 2004 – Dec 2008	**a. HIV + residents not on ART (n = 1,003):** population-based surveillance in 6 catchment areas,	Multivariate logistic regression	**Sex (not associated):** no significant association between gender and receiving treatment: aOR men 0.875 [95% CI: 0.708-1.081, p = 0.216] **Age (associated**, younger (15–19 yrs) < older (>19 yrs)**):** compared to age 15–19 (reference) all higher 5-year-age-groups [20–24, 25–29, 30–34, 35–40, 40–45, 45–50, 50–54, 55–60, >60] have significant higher aOR [ranging between 4.9-14.0, p < 0.05] for receiving treatment **Area of living (not associated):** no significant differences in aORs between peri-urban [1.042, 95% CI: 0.699-1.554, p = 0.838], rural [0.941, 95% CI: 0.628-1.410, p = 0.768] and urban (reference) areas for receiving treatment **Socioeconomic status (not associated):** no significant differences in aORs between index profiles 1 (reference), 2 [0.932, 95% CI: 0.688-1.262, p = 0.649], 3 [0.842, 95% CI: 0.624-1.135, p = 0.258], 4 [0.829, 95% CI: 0.607-1.131, p = 0.237] and 5 [0.984, 95% CI: 0.702-1.379, p = 0.927] for receiving treatment **Education (not associated):** no significant association between years of education and receiving treatment; aOR years of education: 1.022 [95% CI: 0.995-1.063, p = 0.128]
				**b. HIV + residents on ART (n = 1,251):** population based 2008 cohort (HIV+, > 15 yrs, on ART)		
Govindasamy 2011 *** Observational [26]	To assess the proportion and characteristics of individuals who accessed HIV care after testing HIV + in a mobile testing unit	**Rural area:** Cape Metropolitan region, Western Cape province, **type of clinic not clearly reported**	Tested HIV+: 2008–2009. Interviewed: Apr-Jun 2010.	Patients tested HIV + in mobile testing units that: **a. linked to ART care (i.e. receiving CD4 test result), b. not linked,** A random sample of patients tested HIV + between August 2008 – December 2009, ≥18 yrs, CD4 < 350, received CD4 test results, available socio-demographic variables was selected using mobile testing unit records **(n = 77)**	Binomial univariate and bivariate regression analysis	**Sex (not associated):** same likelihood to link to care for female as male patients; **RR female: 1.18 [95% CI: 0.81-1.72, p = not reported, 1.0 falls within CI] **Age (not associated):** same likelihood to link to care for younger (≤30 years) as older patients (≥30 years) to link to care; **RR ≥30 years: 1.21 [95% CI: 0.83-1.77, p = not reported, 1.0 falls within CI] **Severity of disease (contradicting results,** CD4 cell count associated and WHO stages not associated**):** significantly lower likelihood to link to care for patients with high (>350) compared to low (≤350) CD4 cell count;*RR CD4 > 350: 0.49 [95% CI: 0.27-0.87, p = 0.014] / same likelihood to link to care for patients in WHO stage I as WHO stage II, III or IV; **RR WHO clinical stage I: 0.88 [95% CI: 0.65-1.18, p = not reported, 1.0 in CI] **Education (not associated):** same likelihood to link to care for patient completed primary school as patients that have not; **RR completed primary school: 1.17 [95% CI: 0.66-2.08, p = not reported, 1.0 falls within CI] **Employment (not associated**, employed < unemployed**):** likely lower likelihood to link to care for employed compared to unemployed patients; **RR employed: 0.72 [95% CI: 0.51-1.01, p = 0.056]. * = univariate ** = bivariate analysis
Tsai 2009 ** Observational [33]	To assess differences in socioeconomic profiles between those who access HIV-related clinical services and the HIV-infected individuals living in the wider community	**Rural area:** Limpopo province, **public hospital**	Community survey: 2004-2005. Clinic survey: Jan 2003 – Nov 2005	**a. community sample, HIV + not on ART (n = 242):** household survey, random sampled from eight rural villages in the province (14–35 yrs, HIV+),	Uni-variate comparison and multiple regression	**Sex (not associated):** no significant difference percentage women in the community vs. clinic sample: 79% vs. 79% [p = 0.78] **Age (associated,** younger (18–30 yrs) < older (30–35 yrs)**):** significant difference in age distribution between community and clinic sample: 18–20 yrs: 13% vs 3.6%; 21–25 yrs: 33% vs. 16%; 26–30 yrs: 36% vs 33%; 31–35 yrs: 18% vs. 47%; X^2^ = 85 [p < 0.001*] **Education (associated,** higher education > lower education**):** significant difference in distribution educational attainment between community and clinic sample: in clinic less likely to completed secondary education [p < 0.001], but more likely to completed matric or tertiary education [p = 0.04] X^2^ 42 [p < 0.001*] **Employment (associated**, not having salaried employment < having salaried employment, unemployed < employed**):** significant difference percentage having salaried employment between community and clinic sample: 6.2% vs. 11%, X^2^ 3.8 [p = 0.05] and in percentage unemployed and able to work: 57% vs. 37%; X^2^ 26 [p < 0.001*] **Marital status (associated,** never married < married or cohabiting): significant difference distribution marital status between community and clinic sample: never married: 78% vs. 43%; married/ cohabiting: 16% vs. 30%; X^2^ 83 [p < 0.001*] *also significant after multivariable regression
				**b. clinical sample, HIV + on ART (n = 534):** convenience sample of patients (18–35 yrs) in primary HIV/AIDS provider hospital, referred by 45 primary health care clinics. Note: samples were not taken from identical sub-districts		
Adam 2009 ** Observational [34]	To quantify the coverage in South Africa up to the middle of 2008, according to various definitions of antiretroviral treatment eligibility	**Rural and urban:** National/ nine provinces, **public clinics**	2008	For nine provinces: **a. number of HIV + in need for ART**: Markov model on HIV progression using different CD4 count compartments	Comparison ART coverage data	**Area of living (associated,** unequal coverage among nine provinces**):** unequal ART coverage in 2008 among 9 provinces: Eastern Cape 32.4%, Free State 25.8%, Gauteng 43.5%, KwaZulu-Natal 39.4%, Limpopo 32.2%, Mpumalanga 31.2%, Northern Cape 61.1%, North West 35.4%, Western Cape 71.1%
				**b. number of HIV + on ART**: estimates of patients starting ART in public health facilities using Department of Health unpublished internal report (7 May 2009)		
Muula 2007 * Systematic review [24]	To describe the gender distribution of patients accessing ART in Southern Africa	**Rural and urban:** National (1999–2004), Khayelisha township in Capetown (2001–2), Eastern cape province 2001–4), Northern cape province (2001–5), **public clinics**	2000 – 2006	**a. National HIV + prevalence female/male ratio in 2005,**	Comparison female/male ratios	**Sex (associated,** male < female**):** female have higher access than men to ART: HIV prevalence female/male ratio = 1.2, while 4 studies report access to ART female/male ratio of 1.9, 2.3, 1.8 and 1.5
				**b. access to ART female/male ratio.** Sampling methods not reported		
Nattrass 2006 * Critical assessment [23]	To compare ART roll-out in public sector between provinces in 2003-2005	**Rural and urban:** National (nine provinces), **public clinics**	2003 - 2005	For nine provinces:	Comparison ART coverage data	**Area of living** (**associated,** unequal coverage among 9 provinces)**:** unequal ART coverage at the end of 2005 among 9 provinces: Eastern Cape 21.8%, Free State 21.0%, Gauteng 29.6%, KwaZulu-Natal 20.0%, Limpopo 27.3%, Mpumalanga 20.9%, Northern Cape 32.3%, North West 24.5%, Western Cape 55.7%
				**a. number of HIV + in need for ART,**		
				**b. number of HIV + on ART,** estimates of ART coverage based on ASSA2003 demographic model (includes public, NGOs and private sector providers)		

CI = Confidence Interval, aOR = adjusted odds ratio, ART = antiretroviral therapy, WHO = world health organization. * = low quality, ** = medium quality, *** = high quality.

**Table 4:** Overview of findings per study reporting on equity in ART adherence

**Study, quality score, study type**	**Study objective**	**Study area, type of clinic/program**	**Year of data collection**	**Study design (comparison between population a and b), population sizes, sampling method and inclusion criteria**	**Statistical analysis**	**Main outcome of analyzed equity criteria**
Boyles 2011 *** Observational [30]	To determine the factors predicting loss to follow-up and mortality in a public-sector HIV and ART programme in rural South Africa	**Rural area:** Elliotdale/Xora area of Mbhashe sub-district in Eastern Cape province, **combined public/donor program**	Jan 2005 – Sept 2009	**a. HIV + patients that loss to follow up (n = 117 (6.5%)),**	Multiple Cox proportional hazard regression	**Sex (not associated):** females and males have same risk of being loss-to-follow-up: HR female: 1.42 [95% CI 0.90-2.23, p = 0.134] **Age (associated**, younger (<25 yrs) < older (25–50 yrs))**:** younger people have significant higher risk to loss-to-follow-up: HR <25 yrs (compared to 25–50 yrs): 1.87 [95% CI: 1.15-3.05, p = 0.012] **Severity of disease (associated**, ≥ 200 CD4 < <200 CD4**)**: higher CD4 cell count significantly increases risk to loss-to-follow-up: 50–199 CD4 (referent); HR 0–49 CD4: 1.00 [95% CI: 0.61-1.64, p = 0.019]; HR ≥ 200 CD4: 1.74 [95% CI 1.09-2.78, p = 0.019]
				**b. HIV + patients that do not loss to follow up (n = 1686).** Both groups are patients enrolled in clinics of Madwaleni HIV wellness and ART program including adherence counseling and home visits (i.e. Madwaleni Hospital, its 7 primary healthcare feeder clinics and a community based outreach program): tested HIV+, ART naïve at time of study enrollment, >19 years, initiated ART (CD4 < 200 CD4), could be follow for at least 3 months **(n = 1803)**		
Orrell 2003 *** Observational [31]	To determine adherence of an indigent African HIV-infected cohort initiating ART to identify predictors of incomplete adherence and virologic failure	**Urban area:** Cape Town, Western Cape province, **university of Cape Town clinic**	Jan 1996 – May 2001	**a. Patients discontinued 48 weeks of ART (n = 47),**	T-test (age, VL, CD4 cell count), X^2^ test (gender, socioeconomic status)	**Sex (not associated)**: no significant difference in percentage female between those discontinued (40.4%) and completed (43.4%) 48 weeks of ART [p = 0.7] **Age (associated**, younger < older**):** those discontinued ART before 48 weeks were significantly younger (31 yrs) than those completed (34.1 yrs) [p <0.005] **Severity of disease (contradicting results,** CD4 cell count associated and WHO stages not associated**):** those discontinued ART before 48 weeks had significantly lower mean CD4 cell count (197) than those completed (268) [p < 0.01] / those discontinued before 48 weeks ART had a significantly higher VL (5.71 log_10_) than those completed (5.49 log_10_) [p <0.05] / no significant difference in percentage WHO stage 3 or 4 between those discontinued (49.2%) and completed (38.2%) 48 weeks of ART [p = 0.2] **Socio-economic status (not associated**): no significant difference in the percentage of patients with low socio-economic status in the group that discontinued (36.2%) and completed (43.6%) 48 weeks of ART [p = 0.4]
				**b. Patients that completed 48 weeks of ART (n = 242).** Both groups are from Cape Town AIDS Cohort (CTAC): HIV + patients, presenting at University of Cape Town HIV clinics (referred by health care workers in the public sector of the wider Cape town area, mainly serving indigent populations), were ART naïve and eligible for adherence monitoring		
Kranzer 2010 *** Observational [27]	To investigate the frequency and risk factors of defaulting treatment and identify factors associated with subsequent return to care in a long-term treatment cohort in South Africa	**Peri-urban**: township in Cape Town, Western Cape province, **public clinic**	Mar 2004 - Dec 2009	**a. HIV + patients that defaulted ART (n = 291),**	Multivariate Poisson regression	**Sex (associated,** men < women**):** compared to women, men have a significant increased risk to default ART treatment, HR men: 1.51 [95% CI: 1.18-1.93, p < 0.01] **Age (not associated):** no significant association between age and defaulting treatment, compared to younger age (≤30 years), HR > 30 years: 0.90 [95% CI: 0.70-1.15, p = 0.40] **Severity of disease (contradicting results,** CD4 cell count associated and WHO stages not associated**):** higher CD4 cell count increases significantly risk for defaulting treatment, ≤100 CD4 (referent); 101–200 CD4: HR 1.32 [95% CI: 0.99-1.76, p = 0.06], CD4 > 200 HR: 1.39 [95% CI 1.02-1.91, p = 0.04]. No significant difference in the risk of defaulting treatment being in WHO stage 3/4 or 1/2, HR stage 3/4: 1.14 [95% CI: 0.85-1.53, p = 0.37]
				**b. HIV + patients that not defaulted ART (n = 863).** Both groups are from patients presenting at public-sector primary care clinic (single ART server in the area), >15 years, started ART (until 2007 < 350 CD4 cells (NIH research study), after 2007 < 200 CD4 cells (provincial ART program) **(n = 1154)**		
Fatti 2010 *** Retrospective cohort study [29]	To compare clinical, immunological and virological outcomes between rural and urban children on ART in a large cohort from multiple public health facilities in four provinces of South Africa	**Rural and urban**: areas in Western Cape, KwaZulu-Natal, Eastern Cape and Mpumalanga province, **public clinics supported by NGOs**	Nov 2003 – Mar 2008	**a. Children on ART that loss to follow up (n = 179),**	Multivariable Cox proportional hazards regression	**Sex (not associated):** gender is not associated with risk of LTFU: HR male: 1.1 [95% CI: 0.82-3.12, no p value reported, 1.0 falls within CI] **Age (not associated):** younger children (<2 yrs) are as likely to LTFU than older children (>2 yrs): > 2 yrs (referent); HR 1–2 yrs: 1.61 [95% CI: 0.96-2.68, no p value reported, 1.0 in CI > 0.90]; HR < 1 yr: [1.81, 95% CI: 0.94-3.64, no p value reported, 1.0 in CI] **Severity of disease (contradicting results,** CD4 cell count not associated and WHO stages associated**):** severe clinical status is associated with risk LTFU: HR severe clinical status: 1.47 [95% CI: 1.03-2.12, no p value reported, 1.0 not within CI]/ severe immunodeficiency was associated with risk LTFU: HR severe immunodeficiency: 0.81 [95% CI: 0.52-1.24, p value not reported, 1.0 in CI **Area of living (associated**, rural/urban < urban/urban < rural/rural**):** patient in rural areas visiting clinics in urban areas are more likely to LTFU than patients from rural areas visiting rural clinics and patients in urban areas visiting urban clinics: rural (referent); HR urban: 1.14 [95% CI: 0.57-2.24]; HR rural/urban 2.85 [95% CI, 1.41-5.79] [p = 0.004]
				**b. Children on ART that do not loss to follow up (n = 2153).** Both from retrospective cohort of children, (<16 yrs, ART naïve), enrolled in 44 routine public healthcare facilities (7 rural, 33 urban/12 secondary level hospitals, 32 primary health care clinics) supported by a NGO, used electronic data collection systems for patient monitoring. Children were divided in 3 groups a) urban residence and urban ART facility attended (urban group, **n = 1727**); rural residence and rural facility attended (rural group, **n = 228**); and rural residents attending urban facilities (rural/urban group, **n = 377**)		
Cornell 2009 ** Observational [28]	To investigate the impact of gender and income on survival and retention in a South African public sector ART programme	**Urban:** Nyanga township, outskirts of Cape Town, Western Cape province, **public clinics supported by NGOs**	Sept 2002 – Apr 2007	**a. HIV + patients that loss to follow up (n = 137),**	Proportional hazards regression models	**Sex (not associated):** gender is not associated with risk to LTFU: HR men: 1.38, [95% CI: 0.94-2.03, p = 0.100] **Age (not associated):** no significant difference between age and risk to LTFU: HR age: 0.98 [95% CI 0.96-1.00, p = 0.102] **Severity of disease (contradicting results,** CD4 cell count associated and WHO stages not associated**)**: patients with CD4 cell count <50 have higher risk to LTFU than CD4 cell count 50–150, but a similar risk as CD4 > 150: CD4 < 50 (referent); HR CD4 51–100: 0.62 [95% CI: 0.37-1.05, p = 0.077]; HR CD4 101–150 [0.57, 95% CI: 0.33-1.00, p = 0.049]; HR CD4 > 150: 1.01 [95% CI: 0.64-1.59, p = 0.971]/ WHO stage has no association with risk to LTFU: WHO stage I & II (referent); HR stage III: 0.78 [95% CI: 0.50-1.21, p = 0.274] HR stage IV: 0.75 [95% CI 0.75 (0.44-1.28), p = 0.294] /VL was not significantly associated with risk to LTFU: HR RNA level <5 log10 copies/ml (referent); >5 log: 1.13 [95% CI: 0.78–1.64, p = 0.520] **Employment (associated**, no income < income**):** patient with no income have a increased risk to LTFU: HR with income: 0.53 [95% CI: 0.37-0.77, p = 0.002]
				**b. HIV + patients that do not loss to follow up (n = 2059).** Both groups from Gugulethu clinic patient cohort that receive adherence counseling including home visits, >15 years, ART naïve, WHO stage IV or CD4 < 200 **(n = 2196)**		

CI = confidence interval, HR = hazard ratio, ART = antiretroviral therapy, WHO = world health organization, LTFU = loss to follow up, VL = viral load.

* = low quality, ** = medium quality, *** = high quality.

**Table 5:** Overview of quality rating scoring per study

**High (30–40 points)**									**Medium (20–29 points)**			**Low (<20 points)**	
**Studies**													
		**Kranzer[**[27]**]**	**Cooke[**[32]**]**	**Fatti[**[29]**]**	**Govindasamy[**[26]**]**	**Boyles[**[30]**]**	**Cleary[**[25]**]**	**Orrell,[**[31]**]**	**Cornell[**[28]**]**	**Tsai[**[33]**]**	**Adam[**[34]**]**	**Muula[**[24]**]**	**Nattrass[**[23]**]**
**Total score** (out of 40 points)		**37**	**34**	**34**	**33**	**32**	**31**	**30**	**26**	**26**	**26**	**19**	**12**
1	Study design (peer reviewed = 2, other = 0)	2	2	2	2	2	2	2	2	2	2	2	2
2	Well-defined hypothesis/objective/research question? (fully = 2, partial = 1, not at all = 0)	2	2	2	2	2	2	1	2	2	2	2	1
3	Clear motivation research question? (fully = 2, partial = 1, not at all = 0)	2	2	2	2	1	2	2	2	2	2	2	1
4	Concept clearly defined (e.g. access, equity) (fully = 2, partial = 1, not at all = 0)	2	1	1	2	2	1	2	0	1	2	0	0
5	Methods well described? (fully = 2, partial = 1, not at all = 0)	2	2	2	2	2	2	1	1	2	2	2	0
6	Main outcomes clearly described? (fully = 2, partial = 1, not at all = 0)	2	2	2	2	1	2	2	2	1	2	2	1
7	Potential sources of bias taken into account? (fully = 2, partial = 1, not at all = 0)	2	2	2	2	2	2	1	0	0	2	0	0
8	Population and sampling method clearly defined? (fully = 2, partial = 1, not at all = 0)	2	2	2	2	2	2	2	1	1	0	0	0
9	Type of information used (i.e. sample size, time period) clearly described? (fully = 2, partial = 1, not at all = 0)	2	2	2	2	1	2	2	1	2	1	1	1
10	Primary data used for key analyses? (yes = 2, no = 0)	2	0	2	2	2	0	2	2	0	0	0	0
11	Survey (household/provider level) data used? (yes = 2, partial = 1, no = 0)	2	2	2	2	2	1	2	2	0	2	0	0
12	Research/subquestion(s) answered? (fully = 2, partial = 1, not at all = 0)	1	2	2	1	2	2	1	1	2	2	2	1
13	Results based on evidence derived from the data analysis? (fully = 2, partial = 1, not at all = 0)	2	2	2	2	2	1	2	2	2	0	0	1
14	Results credible given the methods, data, and analysis used? (fully = 2, partial = 1, not at all = 0)	2	2	2	2	2	1	1	1	2	2	0	1
15	Robustness of findings and limitations of method discussed? (fully = 2, partial = 1, not at all = 0)	2	2	2	1	1	2	2	1	2	1	2	0
16	Findings discuss within context of existing evidence base? (fully = 2, partial = 1, not at all = 0)	2	2	2	2	2	2	2	2	2	2	2	1
17	Missings clearly described? (fully = 2, partial = 1, not at all = 0)	2	2	1	0	2	1	1	2	0	0	0	0
18	Generalizable to rest of the country? (given sample size) (fully = 2, partial = 1, not at all = 0)	2	1	2	1	1	0	0	0	1	2	2	2
19	Study subjects asked representative of entire population recruited from? (yes = 2, no = 0)	0	0	0	0	0	2	2	2	0	0	0	0
20	Study subjects prepared to participate representative of entire population recruited from? (yes = 2, partial = 1, no = 0)	2	2	0	2	1	2	0	0	2	0	0	0

### Equity in utilization of ART

For sex, two [24],[27] out of ten studies [24]-[33] that reported on this equity criterion found an association between sex and utilization of ART. In both studies (high- and low-quality) men appear to have lower utilization of ART compared to women. The other eight studies (six high- and two medium-quality) found no association [25],[26],[28]-[33].

Four [30]-[33] (three high and one medium-quality) out of eight studies [26]–[33] reported that relatively young people have a lower utilization of ART. The other four studies (three high- and one medium-quality) that reported on age found no association [26]-[29].

For severity of disease, five [26]-[29],[31] out of six studies [26]-[31] reported contradicting results. In four [26]-[28],[31] out of these five studies an association was found between ART utilisation and a person’s CD4 cell level while no association was found with a patient’s WHO status. Of these studies, one ART initiation [26] and one on adherence [27] (both high quality) reported that higher CD4 cell counts are associated with lower utilization of ART. On the contrary, two other studies on adherence (one high- and one medium-quality) reported that lower CD4 cell count is associated with less utilization [28],[31]. In one other study (high quality) that reported contradicting results for severity of disease among children, an association was found with WHO stage but not with CD4 cell count level [29]. The sixth study (high quality) reporting for severity of disease, only looked at CD4 cell count levels and found that patients with a higher CD4 cell count level adhered less to ART [30].

For area of living, three [23],[29],[34] out of the four studies [23],[29],[32],[34] that reported on this criterion found an association between area of living and ART utilization. Two studies (high- and medium-quality) reported that people in certain provinces have lower utilization of ART (see Table 3) [23],[34]. One of the studies (high-quality) reported that children living in rural areas and who visit ART clinics in urban areas, have lower utilization than children that visit clinics in their own area of living (urban or rural area) [29]. The fourth study (high-quality) that reported on area of living found no association between ART utilization and area of living (peri-urban, urban or rural area) [32].

Socioeconomic status was found to be associated with ART utilization in two [28],[33] (both medium-quality) out of the six studies [25],[26],[28],[31]-[33] that reported on this criterion, which showed that those unemployed have lower utilization of ART. One of these two studies also reported that those with lower education utilize less [33]. Of the four studies that found no association, one (high-quality) found no differences on the basis of employment and education [26]. The other three (all high-quality) found no differences in ART utilization between those with differences in SES [25],[31],[32]. One of these also found no association between educational level and ART utilization [32].

For marital status only one study (medium-quality) was included in this review and reported that being unmarried is associated with lower ART utilization [33]. For ethnicity only one study (high-quality) was found, and it reported inconclusive results due to a small sample size [25]. None of the included studies looked at the ART utilization by religion or sexual orientation.

## Discussion

This is the first systematic review that examines equity in utilization of ART in South Africa and identified 12 studies. It seems that men, young people, those living in certain provinces or rural areas, people who are unemployed or with low educational level, or those who are unmarried have less access to ART. For severity of disease, most studies used more than one outcome measure for disease stage and reported within their study contradicting results. No evidence of inequity in ART utilization by ethnicity, religion and sexual orientation was found. There were large heterogeneities in both context (study area, type of program, time period) and methodology of the studies in this review.

Only one high- and one low-quality study reported a significant difference in utilization of ART among men and women, and eight other studies found no differences. Although it is encouraging that access to ART seems mostly equal for both genders, the studies in our review failed to take the timing of ART initiation into account. Observational studies from South Africa recently showed that case-fatality rates among HIV-infected men were substantially higher compared to women in South Africa, most likely related to late entry into care [35],[36]. Late entry by men can be explained as ART is mainly provided through primary health care services, and its antenatal care services frequently serve as an entry- point for HIV treatment for women.

The findings in some studies which showed that young age is associated with low utilization raises concerns. Young people may face more barriers to treatment (like lack of knowledge about treatment possibilities and benefits and fear for stigma and discrimination) [32]. Yet, this relationship may be confounded by eligibility, as older people are more likely to be eligible because of more advanced disease stages. In addition, many studies did not cover all ages. As the HIV epidemic in South Africa is ageing [37],[38] it will become increasingly important to determine ART utilization among elderly, a group previously neglected in research on ART utilization.

Both area of living and SES did not seem to be associated with ART utilization. However, the studies looking at area of living were mostly of low-quality. The studies by Nattrass *et al.*[23] and Adam *et al.*[34] reported coverage levels for different provinces. However, these studies used a simple Markov-model to estimate the need for ART, and it is difficult to determine whether the model projections are valid. The study by Fatti *et al.*[29] reports on children in four different areas. Lower utilization for children living in rural areas and accessing clinics in urban areas can be explained by financial and non-financial barriers such as the monetary cost of transportation or the opportunity cost of accessing health care services [33]. Nevertheless, more research is needed in order to generalize these findings to other areas and population groups. Finally, Tanser *et al.*[39] showed that self-reported visiting of health clinics in a rural South African area was significantly associated with the distance between the clinic and home, with greater distance resulting in lower utilization, yet we did not include this study because it didn’t specifically concern ART utilization.

Studies on SES and area of living will likely measure the same inequities as people in deprived areas might have lower SES. Tsai *et al.*[33] found significant evidence of socioeconomic inequities in the uptake of ART services within a rural and deprived part of South Africa during the early years of the public sector scaling up of ART (2003–2005). Poorer households in South Africa and in sub-Saharan Africa generally have less access because they face various barriers like cost for transport to the clinic, knowledge of the benefit of ART treatment and a lower propensity to seek formal sector treatment for illness [40],[41]. Cleary *et al.*[25] reported no differences in SES distribution between those in need and those accessing ART in urban areas in 2008. This is in line with the ‘inverse equity hypothesis’ which predicts a paradoxical worsening of health inequities as effective new public health interventions first diffuse among the well-to-do but later also among the poor. Last years ART has been scaled-up drastically (and now reaches about 80% of those in need) barriers to access might have been reduced or removed and those least able to overcome those initial barriers are now able to use the services [25]. Yet, still about 20% lacks access to treatment and this group likely faces most barriers. In addition, if South Africa adopts the new WHO guidelines and further expands its ART program new inequities might appear.

We found contradicting results for severity of disease as within studies differences in ART utilization were reported for HIV-infected people with different CD4 cell count levels but not for different WHO disease stages. Also, some studies reported lower utilization for healthier patients while other studies for the most severely ill. One of the studies by Govindasamy *et al.*[26] addressed ART initiation and concluded that those with a CD4 cell count of >350 are less likely linked to care after testing HIV positive than those ≤350. This can be explained by the fact that these patients were not yet eligible for ART and only needed to enrol in the clinic to monitor their CD4 level, or because they feel less need for care as they do not suffer from symptoms. The other five studies addressed ART adherence. Boyles *et al.*[30] and Kranzer *et al.*[27] both found that those with higher CD4 cell count (CD4 > 200) adhere less to ART and this may also be explained by the fact that individuals who default do so because they feel better on treatment [42],[43]. In contrast, Fatti *et al.*[29] and Orrell *et al.*[31] found that most severely ill patients were more likely to lost of follow-up. One explanation could be that patients perceived a lack of effectiveness of treatment when ill or not being able to take the medicine because of symptoms [42]. However, the status of patients who are lost to follow-up is difficult to assess, and it is also likely that many of those are unregistered deaths, thus explaining the higher rates among those with advanced disease.

Only one of the studies looked at marital status and reported less access for unmarried people. However, this study was of medium quality as it compared socio-economic characteristics of a community sample with a clinic sample which were taken from different areas. For ethnicity, religion and sexual orientation no evidence was available and more research is needed to determine inequities in ART utilization by these criteria. It is likely that inequities exist on the basis of ethnicity, as the history of apartheid caused differences in access between black and white South Africans [44]. Also, among black Africans differences in access between ethnic groups like Zulu-speakers, French speaking Cameroonians and Xhosa speakers likely exist, partly due to differences in language barriers that they may face when accessing care [45],[46]. Although HIV-prevention services for men who have sex with men (MSM) are expanding across the country, there are still several gaps [47],[48]. This group may face barriers in ART access due to fear of provider stigma and social isolation [49],[50]. Low HIV testing rates are reported among Muslim people in predominantly Muslim residential areas in Cape Town [51] and different religions might face different levels of HIV-related stigma which might cause inequities in ART utilization [52].

After analyzing the findings of the included studies we found no patterns of equities or inequities that may be explained by differences in program design (e.g. NGO or university supported, public program, availability adherence counselor), time period (e.g. before or after scale up of ART), target population (e.g. indigent populations, children) and study area (e.g. townships, rural areas). On the other hand, patterns might have been identified if the number of studies included in this review were higher.

We found only 12 studies which looked at equity criteria for ART utilization, and two of these were of low- quality. In addition, all studies differed in context (year of study, area, study population), methodology, and outcome measured. Access to ART in South Africa has evolved quickly over the past decade [53] and inequities that were reported at the start of the ART scale-up might no longer be relevant now. Given the incomplete and mixed evidence base, we call for more rigorous analysis on equity of ART treatment in South Africa, and beyond. We flag three important domains. First, reviewed studies were based on different samples and this made any comparison or generalisation difficult to achieve. A national monitoring system on ART initiation and adherence, which also registers key criteria such as severity of disease, gender, age, SES and area of living could fill in this gap. To measure those in need for ART we recommend using the definition ‘eligible for ART on the basis of the country guidelines’ as not all HIV-infected people might be already eligible for ART. Yet, the challenge remains to identify HIV-infected patients who are in need of treatment but have not yet been linked to care. Second, most studies only assessed a few equity criteria. This could be explained by the emphasis in strategic ART plans worldwide to reduce gender, SES and area of living inequities [1]. In addition, the recent *health equity monitor* launched by the WHO uses a list of indicators to present a country’s equity profile, but recommends to differentiate groups on the basis of SES, gender, area of living and education level only [54]. We therefore recommend getting similar insights in inequalities between groups that differ in age, severity of disease, marital status, ethnicity, sexual orientation and religion for ART utilization. Third, studies employed a variety of definitions of both ART initiation and adherence measures, but also of equity criteria measures, indicating the need to develop standardized measures in this area of study.

## Conclusions

On the basis of 12 studies identified in this review it seems that men, young people, those living in certain provinces or rural areas, those who are unemployed or with a low educational level, and those who are unmarried are disadvantaged from utilization of ART. For severity of disease, most studies used more than one outcome measure for disease stage and reported within their study contradicting results. For ethnicity, religion and sexual orientation there was no evidence available to draw conclusions. As studies stem from different contexts and use different methods, findings cannot be generalized and conclusions should be taken with caution. In order to better inform policy makers, we call for improved guidance in equity research on ART, addressing the need to develop national monitoring of inequity of utilization of ART and employing standardized measures of utilization and equity criteria.

## Abbreviations

ART: Antiretroviral therapy

PMTCT: Prevention of mother to child transmission

SES: Socio-economic status

WHO: World Health Organization

## Competing interests

The authors declare that they have no competing interests.

## Authors’ contributions

NT coordinated the study and participated in the design of the study, acquisition, analysis and interpretation of data and helped drafting and finalised the manuscript. CM participated in the design of the study, acquisition, analysis and interpretation of data and drafted the manuscript. EM was involved in the acquisition, analysis and interpretation of the data and revised the manuscript. JH was involved in interpretation of the data and revised the manuscript. RB was involved in the design of the study, helped in interpretation of the data and revised the manuscript. All authors read and approved the final manuscript.

## Additional files

## Supplementary Material

Additional file 1: Database search strategies for systematic review on equity in utilization of ART in South Africa. Click here for file

Additional file 2: Data extraction and quality assessment forms for systematic review on equity in utilization of ART in South Africa. Click here for file
