# Supplementary material for: Equity in utilization of antiretroviral therapy for HIV-infected people in South Africa: a systematic review
Source: Int J Equity Health. 2014 Aug 1;13:60. doi: 10.1186/s12939-014-0060-z (PMC4448289; doi:10.1186/s12939-014-0060-z)
Supplement: Additional file 1: — Database search strategies for systematic review on equity in utilization of ART in South Africa. [file s12939-014-0060-z-S1.docx]

**Additional file 1. Database search strategies for systematic review on equity in utilization of ART in South Africa**

| **Table 1. Pubmed database search on 18 February 2013** | | |
| --- | --- | --- |
| ***Search category*** | ***Syntax*** | ***Results*** |
| **ART** | antiretroviral therapy, highly active[MeSH Terms] OR ART[title/abstract] OR  HAART[title/abstract] OR ARV[title/abstract] OR ARVs[title/abstract] OR  Anti-Retroviral Agents[Mesh] OR antiretroviral[title/abstract] OR anti  retroviral[title/abstract] OR anti-retroviral[title/abstract] OR  antiviral[title/abstract] OR therapy[title/abstract] | 1.251.418 |
| **HIV** | acquired immunodeficiency syndrome[MeSH Terms] OR acquired immunodeficiency syndrome[title/abstract] OR aids[title/abstract] OR hiv[MeSH Terms] OR hiv[title/abstract] OR human immunodeficiency virus[title/abstract] OR hiv infections[MeSH Terms] | 318.414 |
| **South Africa** | (south africa[MeSH Terms] OR (south[title/abstract] AND africa*[title/abstract])) | 41.343 |
| **Equity** | (equity[title/abstract] OR equities[title/abstract] OR inequity[title/abstract] OR inequities[title/abstract] OR equality[title/abstract] OR equalities[title/abstract] OR Equal[title/abstract] OR Equitable[title/abstract] OR inequality[title/abstract] OR inequalities[title/abstract] OR unequal[title/abstract] OR disparity[title/abstract] OR disparities[title/abstract] OR vulnerability[title/abstract] OR fairness[title/abstract] OR unfair[title/abstract] OR social justice[MeSH Terms] OR social justice[title/abstract] OR justice[title/abstract] OR barrier[title/abstract] OR coverage[title/abstract] OR barriers[title/abstract] OR healthcare disparities[MeSH Terms] OR health services accessibility[MeSH Terms] OR health services accessibility[title/abstract] OR access to health care[title/abstract]) | 530.375 |
| **HIV** AND **ART** AND **Equity** AND **South Africa** | Search: (equity[title/abstract] OR equities[title/abstract] OR inequity[title/abstract] OR inequities[title/abstract] OR equality[title/abstract] OR equalities[title/abstract] OR Equal[title/abstract] OR Equitable[title/abstract] OR inequality[title/abstract] OR inequalities[title/abstract] OR unequal[title/abstract] OR disparity[title/abstract] OR disparities[title/abstract] OR vulnerability[title/abstract] OR fairness[title/abstract] OR unfair[title/abstract] OR social justice[MeSH Terms] OR social justice[title/abstract] OR justice[title/abstract] OR barrier[title/abstract] OR coverage[title/abstract] OR barriers[title/abstract] OR healthcare disparities[MeSH Terms] OR health services accessibility[MeSH Terms] OR health services accessibility[title/abstract] OR access to health care[title/abstract]) AND (south africa[MeSH Terms] OR (south[title/abstract] AND africa*[title/abstract])) AND (acquired immunodeficiency syndrome[MeSH Terms] OR acquired immunodeficiency syndrome[title/abstract] OR aids[title/abstract] OR hiv[MeSH Terms] OR hiv[title/abstract] OR human immunodeficiency virus[title/abstract] OR hiv infections[MeSH Terms]) AND (antiretroviral therapy, highly active[MeSH Terms] OR ART[title/abstract] OR HAART[title/abstract] OR ARV[title/abstract] OR ARVs[title/abstract] OR Anti-Retroviral Agents[Mesh] OR antiretroviral[title/abstract] OR anti retroviral[title/abstract] OR anti-retroviral[title/abstract] OR antiviral[title/abstract] OR therapy[title/abstract]) | **297** |

| **Table 2. EMBASE database search on 18 February 2013** | | |
| --- | --- | --- |
| ***Search category*** | ***Syntax*** | ***Results*** |
| **ART** | exp highly active antiretroviral therapy/ OR ART.ti,ab. OR HAART.ti,ab. OR ARV.ti,ab. OR ARVs.ti,ab. OR exp antiretrovirus agent/ OR antiretroviral.ti,ab. OR anti retroviral.ti,ab. OR anti-retroviral.ti,ab. OR antiviral.ti,ab. OR therapy.ti,ab. | 1.704.926 |
| **HIV** | exp acquired immune deficiency syndrome/ OR "acquired immunodeficiency syndrome".ti,ab. OR aids*.ti,ab. OR exp Human immunodeficiency virus/ OR "hiv".ti,ab. OR "human immunodeficiency virus".ti,ab. OR exp Human immunodeficiency virus infection/ | 406.830 |
| **South Africa** | Exp south africa/ OR (south.ti,ab. AND africa*.ti,ab.) | 46.377 |
| **Equity** | equity.ti,ab. OR equities.ti,ab. OR inequity.ti,ab. OR inequities.ti,ab. OR equality.ti,ab. OR equalities.ti,ab. OR "inequality".ti,ab. OR Equal.ti,ab. OR Equitable.ti,ab. OR "inequalities".ti,ab. OR "unequal".ti,ab. OR disparity.ti,ab. OR disparities.ti,ab. OR vulnerability.ti,ab. OR fairness.ti,ab. OR unfair.ti,ab. OR "social justice".ti,ab. OR "justice".ti,ab. OR barrier.ti,ab. OR barriers.ti,ab. OR "health services accessibility".ti,ab. OR "access to health care".ti,ab. OR "coverage".ti,ab. OR exp social justice/ OR exp health care disparity/ OR health care delivery/ | 620.062 |
| **HIV** AND **ART** AND **Equity** AND **South Africa** |  | **433** |

| **Table 3. CENTRAL database search on 18 February 2013** | | |
| --- | --- | --- |
| ***Search category*** | ***Syntax*** | ***Results*** |
| **ART** | (Antiretroviral Therapy, Highly Active OR Anti-Retroviral Agents OR  ART OR HAART OR ARV OR ARVs OR antiretroviral OR anti retroviral OR anti-retroviral OR antiviral OR therapy):ti,ab,kw | 183.638 |
| **HIV** | (acquired immunodeficiency syndrome OR hiv OR hiv infections OR aids OR human immunodeficiency virus):ti,ab,kw | 14.559 |
| **South Africa** | (South Africa OR South Africa*):ti,ab,kw | 1.163 |
| **Equity** | (equity OR equities OR inequity OR inequities OR equality OR equalities OR Equal OR Equitable OR inequality OR inequalities OR unequal OR disparity OR healthcare disparities OR disparities OR vulnerability OR fairness OR unfair OR social justice OR justice OR barrier OR barriers OR health services accessibility OR health services accessibility OR access to health care OR coverage):ti,ab,kw | 21.449 |
| **HIV** AND **ART** AND **Equity** AND **South Africa** |  | **13** |

| **Table 4. PsycINFO database search on 18 February 2013** | | |
| --- | --- | --- |
| ***Search category*** | ***Syntax*** | ***Results*** |
| **ART** | highly active antiretroviral therapy.ti,ab. OR ART.ti,ab. OR HAART.ti,ab. OR ARV.ti,ab. OR ARVs.ti,ab. OR antiretroviral.ti,ab. OR anti retroviral.ti,ab. OR anti-retroviral.ti,ab. OR antiviral.ti,ab. OR therapy.ti,ab. | 182.687 |
| **HIV** | Exp HIV/ OR hiv.ti,ab. OR human immunodeficiency virus.ti,ab. OR exp AIDS/ OR aids*.ti,ab. OR exp acquired immune deficiency syndrome/ OR acquired immunodeficiency syndrome.ti,ab. | 44.968 |
| **South Africa** | south africa or (south and africa*).ti,ab. | 9.086 |
| **Equity** | equity.ti,ab. OR equities.ti,ab. OR inequity.ti,ab. OR inequities.ti,ab. OR equality.ti,ab. OR equalities.ti,ab. OR Equal.ti,ab. OR Equitable.ti,ab. OR inequality.ti,ab. OR inequalities.ti,ab. OR unequal.ti,ab. OR disparities.ti,ab. OR vulnerability.ti,ab. OR fairness.ti,ab. OR unfair.ti,ab. OR social justice.ti,ab. OR justice.ti,ab. OR barrier.ti,ab. OR barriers.ti,ab. OR health services accessibility.ti,ab. OR access to health care.ti,ab. OR "coverage".ti,ab. OR exp social justice/ OR exp health disparities/ OR health care delivery/ equity.ti,ab. OR equities.ti,ab. OR inequity.ti,ab. OR inequities.ti,ab. OR equality.ti,ab. OR equalities.ti,ab. OR Equal.ti,ab. OR Equitable.ti,ab. OR inequality.ti,ab. OR inequalities.ti,ab. OR unequal.ti,ab. OR disparities.ti,ab. OR vulnerability.ti,ab. OR fairness.ti,ab. OR unfair.ti,ab. OR social justice.ti,ab. OR justice.ti,ab. OR barrier.ti,ab. OR barriers.ti,ab. OR health services accessibility.ti,ab. OR access to health care.ti,ab. OR exp social justice/ OR exp health disparities/ OR health care delivery/ | 166.097 |
| **HIV** AND **ART** AND **Equity** AND **South Africa** |  | **58** |
