# Supplementary material for: Equity in utilization of antiretroviral therapy for HIV-infected people in South Africa: a systematic review
Source: Int J Equity Health. 2014 Aug 1;13:60. doi: 10.1186/s12939-014-0060-z (PMC4448289; doi:10.1186/s12939-014-0060-z)
Supplement: Additional file 2: — Data extraction and quality assessment forms for systematic review on equity in utilization of ART in South Africa. [file s12939-014-0060-z-S2.docx]

**Additional file 2. Data extraction and quality assessment form for systematic review on equity in utilization of ART in South Africa**

| **Table 1. Data extraction sheet** | | | | |
| --- | --- | --- | --- | --- |
| **Subject** | **Question** | **Answers** | | |
| **General Information** | Name of person performing data extraction | 1. CM | 2. EM | |
|  | Date of data extraction |  | | |
|  | Author(s) |  | | |
|  | Title article |  | | |
|  | Journal |  | | |
|  | Type of publication | 1. Journal Article, 2. Dissertation,  3. Other: … | | |
|  | Date/Year of publication |  | | |
|  | Geographical study area |  | | |
|  | Source of funding |  | | |
|  | Inclusion criteria study |  | | |
|  | Exclusion criteria study |  | | |
|  | Quality assessment score |  | | |
| **Study Characteristics** | Research question / Objective of the study |  | | |
|  | Study design | 1. Observational 2. Review, 3. Other: | | |
|  | Total duration of the study |  | | |
|  | Source of data and year of data collection? | 1. Demographic health survey 2. Other: …  3. Unclear | | |
|  | Area of interest | 1. Initiation of ART 2. Adherence to ART  3. Other: … | | |
|  | How were participants recruited? |  | | |
|  | Which sampling procedure was used to create a representative sample of the population? |  | | |
|  | Number of participants enrolled in the study (sample size) |  | | |
|  | In what manner was data collected? | 1. Face-to-face interview, 2. Questionnaire,  3. Other… | | |
|  | Compared groups / Number of participants allocated in each group |  | | |
|  | Where was the intervention implemented? (e.g. country level/ hospital/ community clinic/other)? |  | | |
|  | Type of data | 1. Primary, 2. Secondary, 3. Not Clear | | |
|  | What statistical method(s) was used for analysis? |  | | |
|  | Is informed consent correctly handled? | 1. No, 2. Yes, 3. Not mentioned | | |
| **Equity criteria** | Area living (location / setting / place of residency) | 1. Not reported | | 2. Reported |
|  | Race/ethnicity | 1. Not reported | | 2. Reported |
|  | Occupation | 1. Not reported | | 2. Reported |
|  | Age | 1. Not reported | | 2. Reported |
|  | Education | 1. Not reported | | 2. Reported |
|  | Gender | 1. Not reported | | 2. Reported |
|  | Socio economic status (SES) | 1. Not reported | | 2. Reported |
|  | Geographical region | 1. Not reported | | 2. Reported |
|  | Religion | 1. Not reported | | 2. Reported |
|  | Severity of disease (CD4 count / Viral load) | 1. Not reported | | 2. Reported |
|  | Other equity criteria | 1. Not reported | | 2. Reported |
|  | Description of equity criteria used |  | |  |
| **Intervention** | Initiation of ART | 1. Not reported | | 2. Reported |
|  | Adherence to therapy | 1. Not reported | | 2. Reported |
|  | Frequency in use ART | 1. Not reported | | 2. Reported |
|  | Possible / potential care providers |  | | |
|  | By whom was the intervention implemented? | 1. Primary care, 2. Secondary care, 3. Tertiary care | | |
|  | For who was the intervention aimed at? | 1. Urban population  2. Rural population  3. Poor  4. Rich  5. General population | | 6. Sex workers  7. IDU’s  8. MSM  9. Other: …  10. No specific aim |
|  | Was the use of ART free? | 1. Yes, 2. No, 3. Not mentioned | | |
| **Equity criteria** | Equity criterion |  | | |
|  | Definition of criteria mentioned in the study | 1. Not mentioned | | 2. Mentioned |
|  | Number of participants allocated to each group |  | | |
|  | Summary data for each intervention group |  | | |
|  | Notes characteristics equity criterion |  | | |
| **Miscellaneous** | Conclusion about access to ART |  | | |
|  | Comments on equity | e.g. comments on model of care or recommendations authors | | |
|  | Is the outcome generalizable/external vailidity? | 1. Not mentioned | | 2. Mentioned |
|  | Missing participant / Drop outs handled correctly? | 1. Not mentioned | | 2. Mentioned |
|  | Mentioned limitations of the study (like bias etc) | 1. Not mentioned | | 2. Mentioned |
|  | References to other relevant studies |  | | |
|  | Miscellaneous comments by study authors |  | | |
|  | Comments on equity by reviewers (EM / CM) |  | | |
|  | Miscellaneous comments by review authors (EM / CM) |  | | |

| **Table 2. Quality assessment form** | | |
| --- | --- | --- |
| *Subject* | *Questions* | *Score* |
| **Type of publication** | Type study design | Peer-reviewed: 2 /Other: 0 |
| **Research question** | Does the study have a clear and well-defined hypothesis/aim/objective/research question? | Fully: 2, Partial: 1, Not at all: 0 |
|  | Does the study motivate its research question? | Fully: 2, Partial: 1, Not at all: 0 |
| **Concepts** | Does the study clearly define concepts including definitions like access, equity, ART and outcome measures? | Fully: 2, Partial: 1, Not at all: 0 |
| **Methods** | Does the study clearly describe the methods that are used to answer the analytical question(s)? | Fully: 2, Partial: 1, Not at all: 0 |
|  | Are the main outcomes to be measured clearly described in the Introduction or Methods section? | Fully: 2, Partial: 1, Not at all: 0 |
|  | Does the study take potential sources of bias into account? | Fully: 2, Partial: 1, Not at all: 0 |
|  | Does the study clearly define the population and sampling method used? | Fully: 2, Partial: 1, Not at all: 0 |
|  | Is the type of information used in the study in terms of source, sample size, time period, levels etc. clearly described? | Fully: 2, Partial: 1, Not at all: 0 |
|  | Does the study make use of primary (survey) data for its key analyses? | Yes: 2, Not at all: 0 |
|  | Does the study make use of survey (household/provider level) data? | Yes: 2, Partial:1, Not at all: 0 |
| **Data** | Does the study answer (all of) the research (sub)question(s)? | Fully: 2, Partial: 1, Not at all: 0 |
|  | Are results based on evidence derived from the data analysis of the study? | Fully: 2, Partial: 1, Not at all: 0 |
|  | Are the results credible given the methods, data, and analysis used? | Fully: 2, Partial: 1, Not at all: 0 |
| **Goal achievement** | Does the study critically discuss the robustness of findings, potential sources of bias, and possible limitations of the approaches of choice? | Fully: 2, Partial: 1, Not at all: 0 |
| **Findings** | Does the study discuss findings within the context of existing evidence base? | Fully: 2, Partial: 1, Not at all: 0 |
|  | Are the missings / lost to follow up patients clearly described? | Fully: 2, Partial: 1, Not at all: 0 |
|  | Are the results generalizable* given size of the sample of study units? *Generalizability defined as generalizable to the rest of the country | Fully: 2, Partial: 1, Not at all: 0 |
| **Discussion / conclusion** | Were the subjects asked to participate in the study representative of the entire population from which they were recruited? | Yes: 2, Not at all: 0 |
| **Generalizability** | Were those subjects who were prepared to participate representative of the entire population from which they were recruited? | Yes: 2, Partial: 1, Not at all: 0 |
| **Total points** |  | (max 40) |
